# Exposure to normobaric hypoxia shapes the acute inflammatory response in human whole blood cells in vivo

**DOI:** 10.1007/s00424-024-02969-2

**Published:** 2024-05-07

**Authors:** Tina Schönberger, Marie Jakobs, Anna-Lena Friedel, Tina Hörbelt-Grünheidt, Bastian Tebbe, Oliver Witzke, Manfred Schedlowski, Joachim Fandrey

**Affiliations:** 1 Institute of Physiology, University Duisburg-Essen, University Hospital Essen, Hufelandstr. 55, 45147 Essen, Germany; 2 Institute of Medical Psychology and Behavioral Immunobiology, University Hospital Essen, 45147 Essen, Germany; 3 Department of Nephrology, University Hospital Essen, 45147 Essen, Germany; 4 Department of Infectious Diseases, West German Centre of Infectious Diseases, University Hospital Essen, 45147 Essen, Germany; 5 Department of Clinical Neuroscience, Osher Center for Integrative Medicine, Karolinska Institutet, 171 77 Stockholm, Sweden

**Keywords:** Hypoxia, Inflammation, Whole blood cells, Human, In vivo

## Abstract

Cells of the immune defence, especially leukocytes, often have to perform their function in tissue areas that are characterized by oxygen deficiency, so-called hypoxia. Physiological hypoxia significantly affects leukocyte function and controls the innate and adaptive immune response mainly through transcriptional gene regulation via the hypoxia-inducible factors (HIFs). Multiple pathogens including components of bacteria, such as lipopolysaccharides (LPS) trigger the activation of leukocytes. HIF pathway activation enables immune cells to adapt to both hypoxic environments in physiological and inflammatory settings and modulates immune cell responses through metabolism changes and crosstalk with other immune-relevant signalling pathways. To study the mutual influence of both processes in vivo, we used a human endotoxemia model, challenging participants with an intravenous LPS injection post or prior to a 4-h stay in a hypoxic chamber with normobaric hypoxia of 10.5% oxygen. We analysed changes in gene expression in whole blood cells and determined inflammatory markers to unveil the crosstalk between both processes. Our investigations showed differentially altered gene expression patterns of HIF and target genes upon in vivo treatment with LPS and hypoxia. Further, we found evidence for effects of hypoxic priming upon inflammation in combination with immunomodulatory effects in whole blood cells in vivo. Our work elucidates the complex interplay of hypoxic and inflammatory HIF regulation in human immune cells and offers new perspectives for further clinical research.

**Supplementary Information:**

The online version contains supplementary material available at 10.1007/s00424-024-02969-2.

## Introduction

The immune system governed by a complex network of cells and signalling molecules acts as a defence mechanism against infections. In particular, white blood cells must adapt to varying oxygen levels, which can significantly impact their function [4]. In inflammatory conditions like sepsis, where leukocytes face challenges from both hypoxia and infection, immune cell adaptation to inadequate oxygen supply becomes crucial [8]. Hypoxia significantly influences leukocyte function in both physiological and pathological settings by altering leukocyte function and fate with the potential for dysfunctional immune responses and harm to tissues [4, 61].

The transcription factor hypoxia-inducible factor (HIF) plays a central role in adaptation to hypoxia, regulating oxygen-dependent genes to enhance numerous physiological processes [24, 60]. Thereby, HIFs regulate the majority of oxygen-dependent genes and induce genes for enhanced erythropoiesis, iron transport, vascular growth, nitric oxide synthesis, phosphate metabolism, glycolysis and gluconeogenesis [11, 18, 49, 54, 55].

The transcription factor complex HIF-1, comprising oxygen-regulated α-subunits and a constitutively expressed nuclear β-subunit, orchestrates this response. It is of note that regulation of HIF-1 follows a post-translational manner [32, 45]. In normoxic conditions, HIF-α subunits are hydroxylated by prolyl 4-hydroxylase domain (PHD) hydroxylases and degraded by the proteasome [17, 32]. Thereby, the activity of the three homologous hydroxylases PHD1, PHD2 and PHD3 is strictly dependent on the oxygen concentration, which is why they are considered to be the oxygen sensors of the cells [3, 13]. Under oxygen starvation, HIF-α subunits accumulate, translocate into the nucleus and activate HIF-dependent target genes [17, 60].

To counteract oxygen deprivation and thus guarantee cell survival, various strategies increase oxygen availability and delivery. Erythropoietin (EPO) stimulates red blood cell production, while vascular endothelial growth factor (VEGF) promotes angiogenesis [15, 16, 21, 55]. Adrenomedullin (ADM) induces vasodilation, angiogenesis and cell proliferation [5, 7, 46, 63]. Cellular adaptation involves enhanced anaerobic glycolysis, increased glucose uptake mediated by increased expression of glucose transporter 1 (GLUT-1) and the key metabolic enzyme pyruvate dehydrogenase kinase 1 (PDK-1) in parallel to inhibition of oxidative phosphorylation [11, 54].

In leukocytes, inflammatory mediators activate the HIF pathway, further driving gene activation in hypoxia. Pathogens, cytokines, chemokines and environmental changes can directly or indirectly activate HIF-dependent gene regulation [9]. Inflammatory cytokines like tumour necrosis factor alpha (TNF-α) have been shown to induce HIF-dependent gene expression through pathways involving phosphatidylinositol 3‐kinase (PI3K) and nuclear factor kappa-light-chain-enhancer of activated B cells (NF-κB) [26, 28].

Exogenous substances, such as LPS, can induce a cellular HIF response, serving as a model for studying inflammatory conditions. It is known from myeloid cells that LPS increases expression of HIF1A mRNA, mediated by LPS-binding to Toll-like receptor 4 (TLR4) and NF-κB-dependent signalling involving p44/42 mitogen-activated protein kinase (MAPK) [22, 23]. The crosstalk between NF-κB and HIF is reciprocal: The *HIF1A* gene contains binding sites for NF-κB, and NF-κB is important for basal HIF-1 transcription, linking these two master regulatory pathways of innate immunity and hypoxia adaptation [59].

The reciprocal crosstalk between NF-κB and HIF emphasizes their interconnected roles in innate immunity and hypoxia adaptation during inflammation.

The interaction between hypoxia and inflammation, termed inflammatory hypoxia, plays a pivotal role in various diseases such as malignancies, chronic obstructive pulmonary diseases and sepsis [44]. In sepsis patients, the hyperinflammatory phase is characterized by an overexpression of HIF-1, while the immunotolerant phase is marked by reduced HIF-1 levels, contributing to immune dysfunction [19, 20, 50]. These findings suggest that hypoxia signalling holds significant therapeutic potential for manipulating and treating inflammatory diseases, including high-altitude illnesses and sepsis, once the mutual impacts are clearly defined [12]. It should be emphasized that nearly all discoveries thus far have been made in experimental animals, in vitro settings or under pathological conditions in patients. Consequently, it remains challenging to ascertain whether these processes can be directly extrapolated to physiological conditions in vivo among healthy individuals.

However, it is not clear how and to what extent oxygen deprivation and inflammatory responses interact in vivo in human blood cells. To elucidate the complex interplay of hypoxic and inflammatory HIF regulation in vivo and to provide new perspectives for further clinical research, we conducted a human subject study combining both stimuli. We investigated the impact of in vivo hypoxia (10.5% oxygen in the air) on HIF pathway activation in human blood before and after injection of immunostimulating *Escherichia coli* LPS as a model of acute infection. To assess the effect of hypoxic priming, i.e. hypoxic exposure prior to the LPS stimulus, and the effect of immunostimulation on the hypoxic response, we determined blood and vital parameters.

## Methods

### Study design and study population

The study was designed as a randomised, double-blind, crossover trail. Healthy male subjects were exposed to 4 h of normobaric hypoxia at 10.5% oxygen in the air following either LPS stimulation (HOX-LPS) of the immune system (intravenous injection of *E. coli*, HOK364; 0.4 ng LPS per kg body weight) or administration of LPS before 4 h of normobaric hypoxia (LPS-HOX). Transfer time between the two phase of the study (4 h after LPS injection and 4 h of hypoxic exposure) was half an hour.

The study population consisted of 30 healthy male participants (mean age 25.8 ± 2.9 years), with a CRP level below 2 mg/l. Regular use of medication, a body mass index (BMI) greater than 30 and a confirmed COVID-19 infection were the exclusion criteria. Participants of both groups (i.e. HOX-LPS and LPS-HOX) did not differ at baseline levels in age, body mass index, school education, blood pressure, heart rate or white blood cell (WBC) count (Table 1). All volunteers underwent an extensive physical and psychiatric assessment (self‐reported questionnaires and interviews about their medical history) along with a full blood examination, performed and subsequently evaluated by physicians of the Institute of Physiology at the University Medicine Essen. Written and informed consent was obtained from each enrolled individual human subject. Further official approval was obtained from the responsible Ethics Committee of the University Hospital Essen (No 18-8258-BO). The study was conducted according to the principles of the Declaration of Helsinki.

**Table 1:** Sociodemographic, physiological and immunological characteristics of both study groups at baseline. Data are shown as mean ± standard deviation (SD). Differences between groups were analysed by Mann–Whitney *U* and chi^2^ test, respectively. No significant differences were detected (all *p* > 0.05). BMI, body mass index; WBC, white blood cells; LPS, lipopolysaccharide; HOX, hypoxia

	**LPS-HOX** (*n* = 15)	**HOX-LPS** (*n* = 15)
Age (years)	26.07 ± 3.49	25.67 ± 2.9
BMI (kg/m^2^)	24.61 ± 2.57	23.84 ± 1.69
School education (> 12 years)	100%	100%
Blood pressure diastolic (mmHg)	81 ± 6.04	82.67 ± 6.51
Blood pressure systolic (mmHg)	134.33 ± 9.42	133 ± 8.41
Heart rate (bpm)	78.47 ± 8.14	80.13 ± 17.03
WBC count	5622.22 ± 955.43	5086.67 ± 1350.98

### Study conduct

**Fig. 1:**
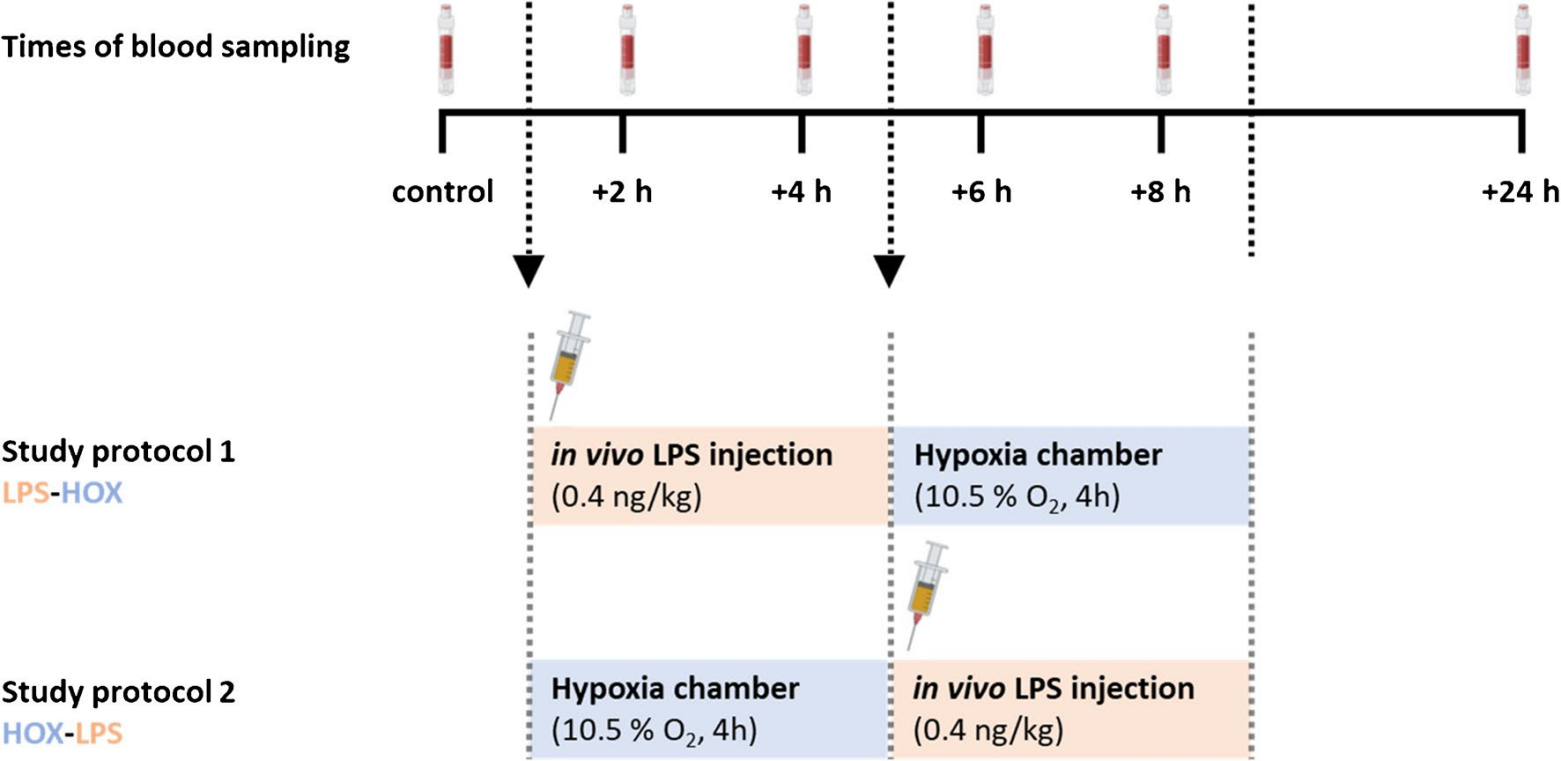
Study design and blood sampling times. Schematic illustration of the two different study protocols and corresponding blood sampling times. Blood samples were taken at six different time points (baseline, + 2, + 4, + 6, + 8 and + 24 h). The study included study protocols with human subjects being treated with a single lipopolysaccharide (LPS) injection in combination with 4 h exposure to 10.5% O_2_ hypoxia before (HOX-LPS) or after the injection (LPS-HOX)

Participants were challenged with a single injection of LPS (*Escherichia coli*, HOK364, 0.4 ng LPS per kg body weight) before or after a 4-h stay in a hypoxia chamber, simulating a high altitude of 4500 m with normobaric oxygen concentration of 10.5% O_2_. Thereby, the subjects were exposed to a reduced fraction of oxygen in the inspiratory gas (10.5%) which is half of the oxygen fraction in air. Before LPS injection, oxygen saturation was controlled to be above 95%. In addition to blood sampling at baseline and follow-up, blood was taken on both study days to determine immunological and hypoxic parameters (Fig. 1). On the study days, a butterfly infusion set was used for venous blood sampling and LPS injection. Blood samples were collected 1 h before the first stimulus (depending on the protocol: the injection with LPS or the stay inside the hypoxic chamber) and 2, 4, 6, 8 and 24 h after the start of the respective study protocol (Fig. 1). Blood samples were immediately processed for gene expression analysis. Blood oxygenation was measured with a finger pulse oximeter throughout the study. A final medical examination was performed 1 week after LPS application. The participants tolerated the procedure well as expected. The LPS injection triggered an increase in body temperature (mild fever), which was accompanied by common side effects like headache and mild shivering in some subjects. Exposure to hypoxia caused mild freezing and tiredness in some participants. All participants were at a stable state of health during the whole study protocol.

### White blood cell count

For the assessment of the WBC count, blood samples were collected every 2 h (+ 2, + 4, + 6, + 8, + 24 h). These samples were then stored in EDTA monovettes at room temperature (RT) until additional processing. WBC count was measured using an automated three-part differential haematology analyser (XP-300, Sysmex).

### Collection of whole blood cells

For direct stabilisation of RNA of whole blood cells, the PAXgene® blood RNA collection system (Qiagen, Mississauga, Canada) was used. Of whole blood, 2.5 ml was drawn into PAXgene® blood RNA tubes and transferred to − 80 °C after storage at RT for 24 h, according to the manufacturer’s protocol.

### RNA preparation and qRT-PCR

RNA of whole blood samples was isolated using the PAXgene® blood RNA Kit (Qiagen, Mississauga, Canada) according to the manufacturer’s protocol.

Real-time PCR (RT-PCR) with SYBR green fluorescent dye (Eurogentec, Verviers, Belgium) and the CFX96TM Real-Time System (Bio-Rad Laboratories GmbH, Munich, Germany) were used for quantification of genes. We reverse-transcribed 100 ng of total RNA into cDNA, which was amplified by 40 cycles of 95 °C for 15 s and 60 °C for 90 s with gene-specific primers (Supplementary Table) and normalized to *ACTB* (actin). Primer specificity was checked by Primer-BLAST and confirmed by size analysis of the PCR amplicons [56]. Expression was normalized to the respective untreated control of each blood donor individually and calculated with the 2 − ΔΔCT method for statistical analysis and set as an induction relative to the respective controls.

### Statistical analyses

Statistical analyses were performed using GraphPad Prism 8.4.3 software (GraphPad, San Diego, CA) and PASW statistics (version 29; SPSS). All values are reported as the mean ± standard deviation. Subject data did not fulfil Gaussian distribution at several time points (BMI, school education) with low sample size, non-parametric calculations were used. Sociodemographic (age, BMI, school education) characteristics were compared between the two study groups using Mann–Whitney *U* and chi^2^ test. All gene expression data sets were analysed as repeated measurements of each single participant over time. Data sets were checked for Gaussian distribution of data points via statistical testing via the Anderson–Darling, D’Agostino-Pearson omnibus and Shapiro–Wilk test. Additionally, Gaussian distribution was confirmed via the QQ plot and analysed using the mixed-effects model. Non-Gaussian distributed data sets were transformed to Gaussian distributed data and analysed accordingly. For data comparison within treatment groups, Tukey’s multiple comparisons test was used. For data comparison between treatment groups, Sidak’s multiple comparisons test was used; Statistical significance is displayed as * = *p* < 0.05, ** = *p* < 0.01, *** = *p* < 0.001 or **** = *p* < 0.0001.

## Results

### Hypoxic priming leads to upregulation of LPS-induced body temperature and affects blood pressure in healthy men

Hypoxia — with or without the injection of LPS — expectedly led to changes in oxygen saturation. The injection of LPS increased the body temperature throughout the study protocol.

Exposure to hypoxia significantly reduced blood oxygen saturation at 1 to 4 h compared to baseline levels reaching an average SpO_2_ of 81.9 (± 0.7)%, below normal values of 98.8 (± 0.1)%, equivalent to about 50 mmHg or 6.7 kPa [6]. LPS treatment did not alter oxygen saturation. Notably, after hypoxic priming, oxygen saturation had returned to normal values before LPS administration.

**Fig. 2:**
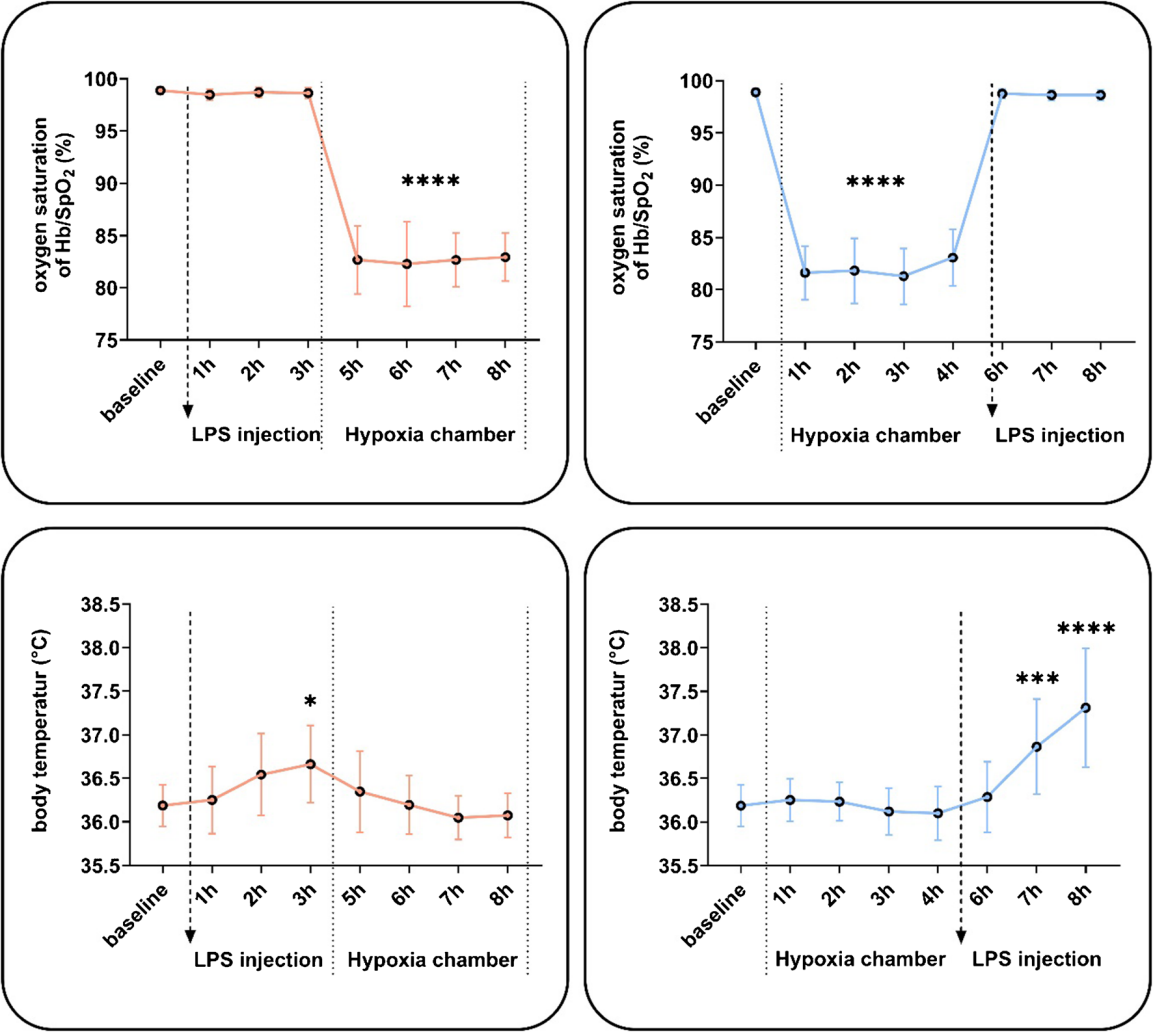
Effect of hypoxia and LPS administration on blood oxygen saturation and body temperature. Oxygen saturation significantly decreased in the hypoxia chamber and decreased under hypoxia after LPS injection. Oxygen saturation remained unchanged after LPS administration with or without a prior hypoxic phase (10.5% O_2_, normobaric). Hypoxia itself had no effect on body temperature, while LPS injection significantly increased body temperature 3 h afterwards. Participants with a hypoxic phase prior to the LPS stimulation showed an even more significant increase in body temperature and inflammatory response. Data are means ± SD. Mixed-effects analysis; *p < 0.05, ***p < 0.001, ****p < 0.0001; *n* = 13–15

Hypoxic exposure did not affect body temperature in healthy men, while LPS treatment resulted in a significant temperature increase at + 3 h post-injection compared to baseline levels. Interestingly, LPS administration after hypoxic priming induced a more pronounced temperature increase at 2 h and 3 h post-LPS injection compared to LPS controls before the hypoxic phase (Fig. 2).

### Effect of HOX-priming on LPS-induced changes in blood leukocytes in healthy men

Exposure to hypoxia had no impact on WBC count while LPS treatment resulted in a statistically significant increase in WBC at + 4 h post-injection compared to baseline levels (baseline 5622 ± 955 vs. LPS treatment 10.967 ± 1143, *n* < 0.0001). However, hypoxic priming did not alter the LPS-induced increase in WBC compared to LPS treatment alone (LPS treatment alone 10.967 ± 1143 vs. HOX-LPS 9953 ± 2276).

### In vivohypoxic and inflammatory priming causes distinct changes in gene expression of whole blood cells

**Fig. 3:**
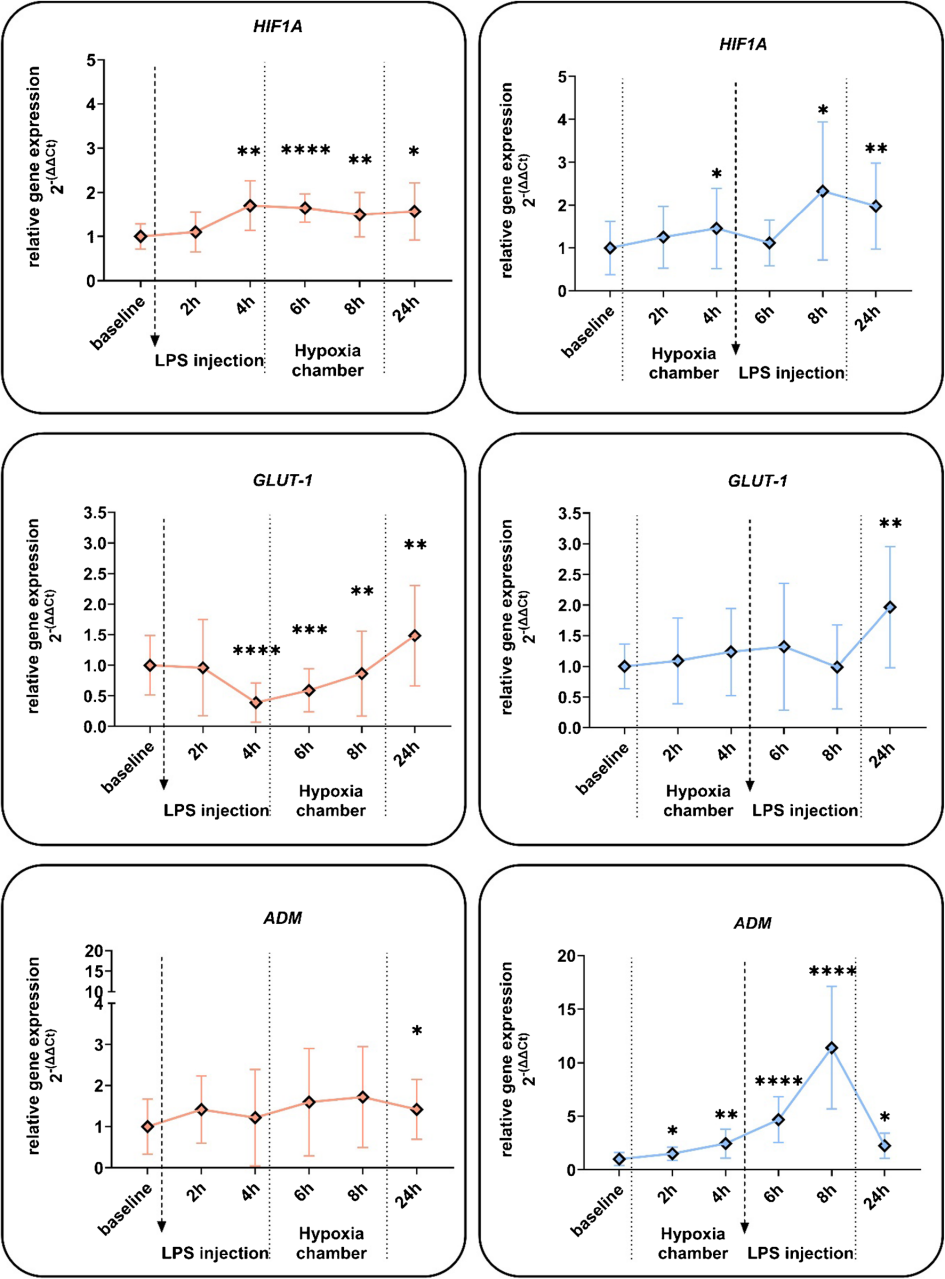
Effects of hypoxia and LPS on HIF and target gene expression of whole blood cells. Relative gene expression of whole blood cells, from blood samples of human subjects that were treated with a single *E. coli* lipopolysaccharide (LPS) injection (0.4 ng/kg) and a 4-h phase of exposure to hypoxia (normobaric, 10.5% O_2_) prior or after LPS injection. Shown are mRNA expression fold changes from hypoxia-inducible factor 1α (*HIF1A*), glucose transporter type 1 (*GLUT-1/SCLA2*) and adrenomedullin (ADM). Expression levels of mRNA were normalised to *ACTB* and are presented as 2 − (ΔΔCT) values (mean ± SD, mixed-effects analysis with repeated measures and Tukey’s or Sidak’s multiple comparisons test; *p < 0.05, **p < 0.01, ***p < 0.001, ****p < 0.0001, *n* = 8–13)

For the first time, we show that LPS injection induced a significant upregulation of HIF-1α mRNA in human blood cells within 4 h. Likewise, exposure to normobaric hypoxia (10.5% oxygen) induced HIF-1α mRNA at 4 h (Fig. 3). HIF-1α mRNA stayed significantly elevated in all three groups until 24 h. Further, the mRNA of the HIF-1 target gene glucose transporter 1 (*GLUT-1*) was significantly decreased 4 h after LPS treatment (also when followed by hypoxia), but not in subjects exposed to 4 h of hypoxic priming before LPS injection. In both groups, LPS-hypoxia and hypoxia-LPS mRNA for *GLUT-1* remained significantly increased after 24 h (Fig. 3). Adrenomedullin (*ADM*) expression was not changed after LPS followed by hypoxia. However, hypoxic priming boosted the LPS effect three- to sevenfold (Fig. 3). Hypoxia-inducible factor 2α and other typical HIF targets such as pyryuvate-dehydrogenase-1 (*PDK1*) and vascular endothelial growth factor (*VEGF*) were not affected within the 8-h study duration (Supplemental Figure).

**Fig. 4:**
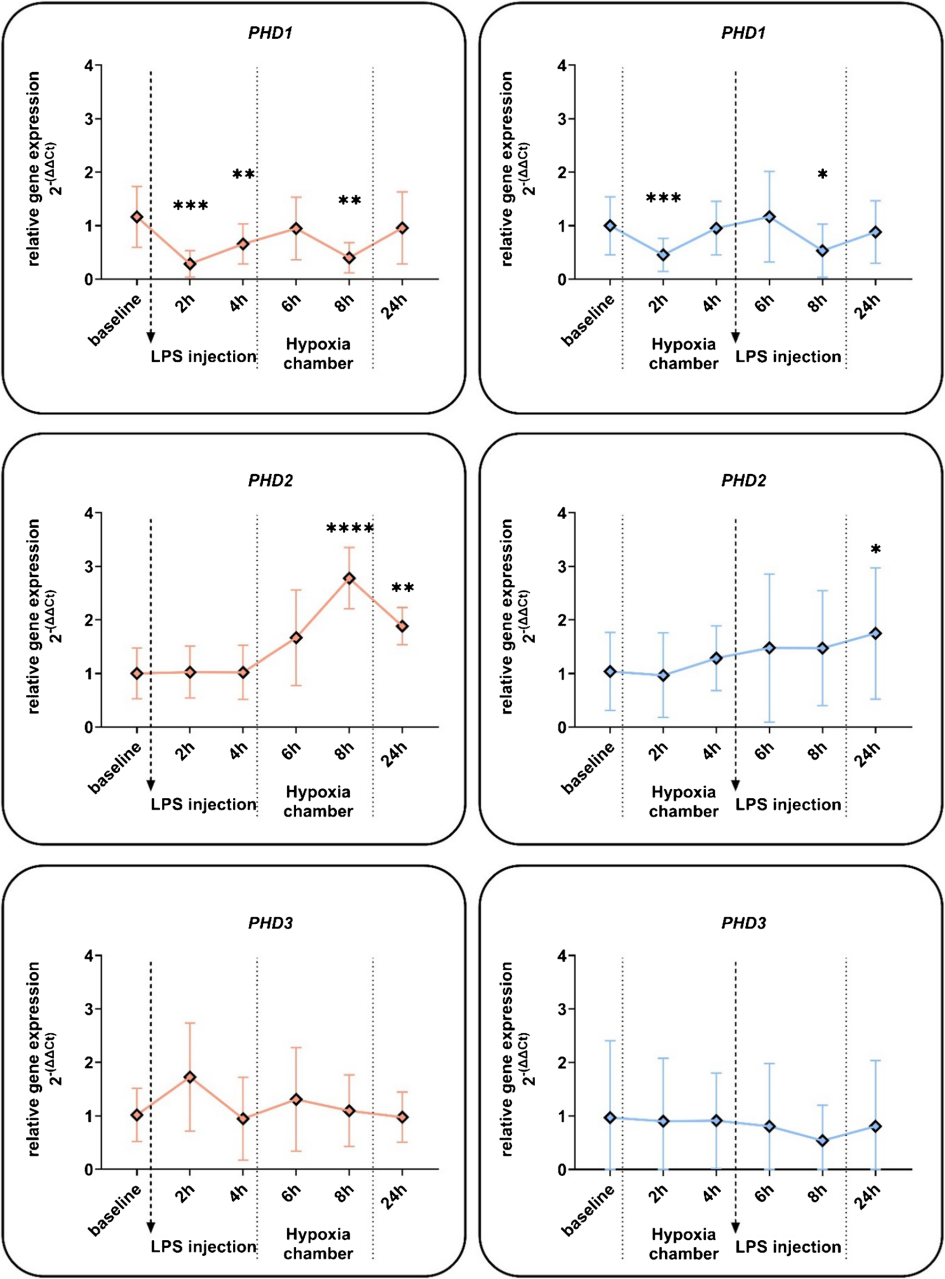
Effects of hypoxia and LPS on the PHD gene expression of whole blood cells. Relative gene expression of whole blood cells from blood samples of human subjects that were treated with a single *E. coli* lipopolysaccharide (LPS) injection (0.4 ng/kg) and a 4-h phase of exposure to hypoxia (normobaric, 10.5% O_2_) prior or after LPS injection. Shown are mRNA expression fold changes from prolyl hydroxylases 1–3 (*PHD1, PHD2, PHD3*). Expression levels of mRNA were normalised to *ACTB* and are presented as 2 − (ΔΔCT) values (mean ± SD, mixed-effects analysis with repeated measures and Tukey’s or Sidak’s multiple comparisons test; *p < 0.05, **p < 0.01, ***p < 0.001, ****p < 0.0001; *n* = 8–13)

Since some prolyl-hydroxylases (*PHD*) are HIF-1 targets, we determined mRNA regulation in whole blood. While *PHD3* showed no significant changes, hypoxia as well as subsequent LPS in the HOX-LPS group led to significant reduction of *PHD1* already after 2 h of hypoxia or 4 h LPS (Fig. 4). Interestingly, the response pattern of both groups (HOX-LPS vs. LPS-HOX) was similar independent of the order of stimuli. *PHD2* mRNA upregulation was unaffected by hypoxic priming, but the hypoxic increase after prior LPS treatment (LPS-HOX group) became highly significant compared to baseline (Fig. 4).

**Fig. 5:**
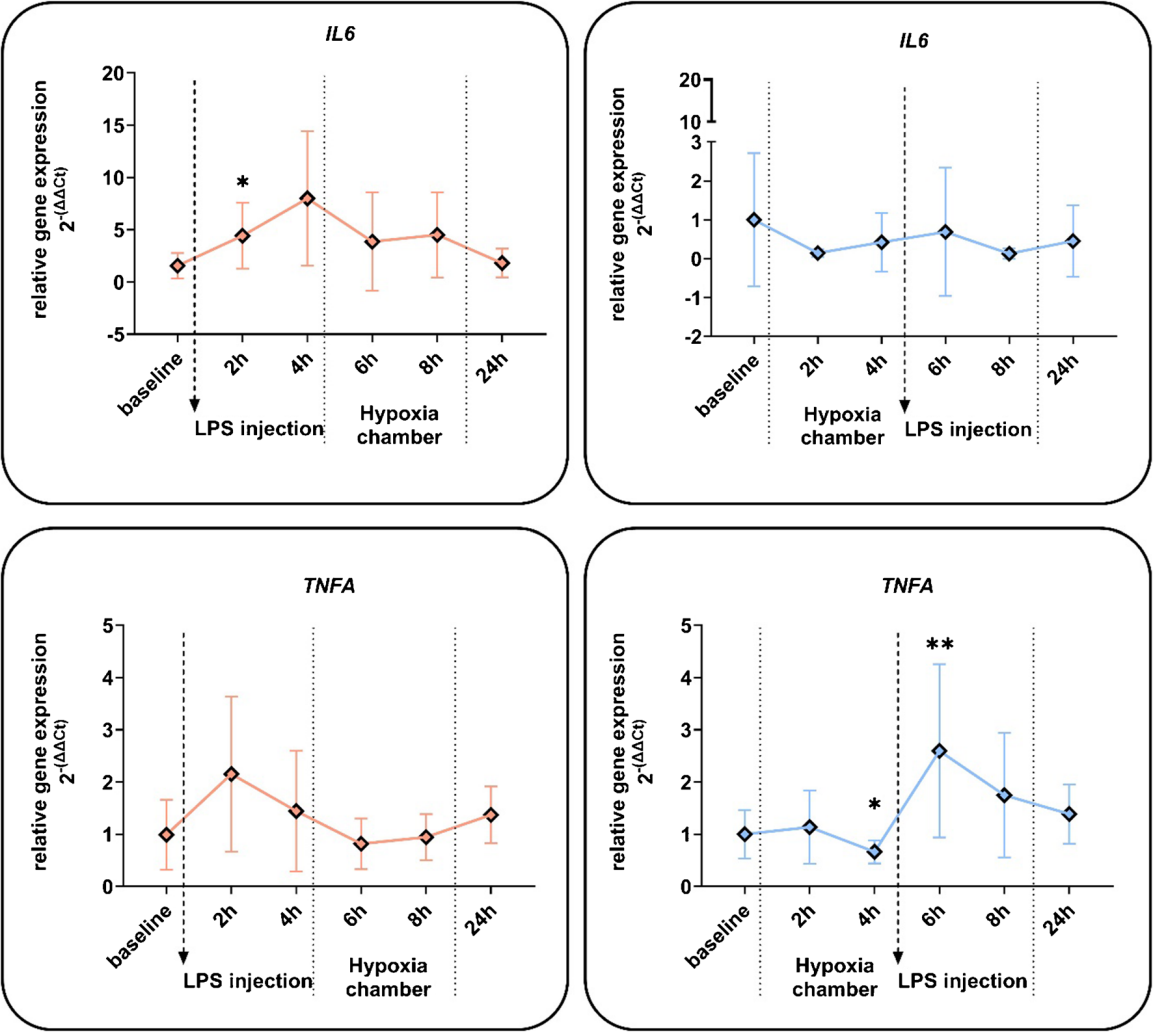
Effects of hypoxia and LPS on the inflammatory gene expression of whole blood cells. Relative gene expression of whole blood cells, from blood samples of human subjects that were treated with a single *E. coli* lipopolysaccharide (LPS) injection (0.4 ng/kg) and a 4-h phase of exposure to hypoxia (normobaric, 10.5% O_2_) prior or after LPS injection. Shown are mRNA expression fold changes from interleukin 6 (*IL6*) and tumour necrosis factor alpha (*TNFA*). Expression levels of mRNA were normalised to *ACTB* and are presented as 2 − (ΔΔCT) values (mean ± SD, mixed-effects analysis with repeated measures and Tukey’s or Sidak’s multiple comparisons test; *p < 0.05, **p < 0.01; *n* = 8–13)

LPS increased the expression of the inflammatory cytokine Interleukin 6 (*IL6*) significantly, with a similar trend in tumour necrosis factor-alpha (*TNFA*). Interestingly, hypoxic priming led to a significant decrease of *TNFA* after 4 h but increased the following LPS effect (6 h time point). The expression of *IL6* was no longer increased when LPS was administered after hypoxia (Fig. 5).

## Discussion

In our study, we investigated the crosstalk of hypoxic and inflammatory stimuli on peripheral blood cells in human subjects using an experimental endotoxemia model [1]. To our knowledge, this was the first study investigating possible in vivo hypoxic priming effects on immune activation. We used a cross-over protocol where healthy young males were challenged with an in vivo intravenous injection of lipopolysaccharide (LPS *E. coli* HOK364, 0.4 ng/kg) before or after exposure to in vivo hypoxia, simulated at an altitude of 4500 m (normobaric, 10.5% O_2_, 4 h).

Typical and well-studied symptoms with an onset 2 h after the in vivo LPS injection can include flu-like symptoms with fever, myalgia and headache [1, 36–38]. This was consistent with our observations. Exposure to hypoxia on the other hand is less studied under normobaric conditions but more under longer-lasting high-altitude (hypobaric) hypoxia [27, 41, 42]. Up to date, no other study focused on the effects of LPS-induced immune activation regarding the HIF pathway nor the potential influence of hypoxic priming on immune cell regulation. To close that gap, we analysed vital parameters of study participants and gene expression in peripheral blood cells.

### Effects of LPS injection and in vivo hypoxia on blood oxygen saturation body temperature

Upon the challenge of participants with LPS, we observed elevated body temperature and increase in white blood cell counts, which all fits to previous reports in the literature [47, 53]. In vivo exposure to hypoxia leads to a reduction in blood oxygen saturation (SpO_2_) but no changes in body temperature.

Following a 3-h exposure to hypoxia, blood oxygen saturation declined from 98.8% to a mean of 83.1% corresponding to approximately 55 mmHg or 7.35 kPa [6]. Oxygen tension varies broadly within the human body, ranging from approximately 80 to 100 mmHg in arterial blood to tissue levels of 9–19 mmHg under physiological conditions. In most cells, the acute hypoxic response mediated by HIF activation will start at levels lower than the recorded PO_2_ values of around 55 mmHg corresponding to the above-mentioned measurements of SpO_2_ under hypoxia. Nevertheless, we identified significant effects on gene regulation in peripheral blood cells supporting our hypothesis of potential hypoxic priming effects even after short-term hypoxia [4, 6]. Obviously, hypoxia as applied in our protocol will lower PO_2_ values in all organs, which are passed by circulating leukocytes, and it is conceivable that leukocyte HIF accumulation will also take place during tissue passage. Moreover, HIF activation will also begin in the parenchyma of peripheral organs to release HIF-dependent gene products such as cytokines or growth factors.

Hypoxic priming has been shown to have complex effects on the immune response, particularly in the context of LPS-induced inflammation. Hempel et al. found that hypoxia can increase the release of the inflammatory cytokines IL-1 and TNF, potentially due to decreased prostaglandin E2 synthesis [29]. This suggests that hypoxic priming may exacerbate the pro-inflammatory effects of LPS. However, the specific mechanisms underlying this interaction are not yet fully understood.

### Gene expression changes in whole blood upon in vivo exposure to hypoxia and LPS immunostimulation

Upon LPS injection, the mRNA levels of HIF1A in whole blood cells exhibited a significant increase 4 and 24 h post-LPS injection confirming previous reports from in vitro studies investigating the impact of LPS injections [50]. This increase in HIF-1A mRNA may have long-term effects on HIF activation in whole blood cells and leukocytes and boosts the cellular response to hypoxia when HIF protein becomes stable.

The expression of the HIF target gene *ADM* showed an interesting picture in whole blood cells. ADM is a multifaceted peptide that plays a significant role in the inflammatory response. It is produced and secreted by peripheral blood monocytes, and monocyte-derived macrophages, with its secretion increasing as these cells differentiate [35]. Furthermore, ADM production is stimulated by cytokines, growth factors and LPS in vascular smooth muscle cells, suggesting its involvement in endotoxin shock, atherosclerosis and inflammation [58]. *ADM* expression is most likely linked to HIF regulation with some independent regulatory mechanisms via HIF-1 and NF-κB through IL-1β as shown in in vitro models [22, 28, 30, 62]. Moreover, previous data on *ADM* expression in patients with sepsis had revealed a correlation between reduced *HIF1A* levels and lower *ADM* expression in peripheral leukocytes [50]. Herein, significant stimulation of *ADM* expression by LPS required hypoxic priming (Fig. 3). Thereby, it could be possible that hypoxic priming directly boosted the LPS effect or that the effect of the hypoxia is prolonged up to 8 h and becomes apparent only after a longer amount of time. Against a delayed reaction argues the fact, which hypoxic response mechanisms need to be and are known to be very fast-acting to ensure sufficient adaption to oxygen shortage. Nevertheless, even hypoxia alone led to a significant increase in *ADM* expression. Currently, it is unclear why LPS without prior hypoxia did not increase ADM mRNA despite elevated HIF1A mRNA levels.

In contrast, the HIF target *PHD2* showed the expected amplification by LPS for the subsequent hypoxic stabilisation of HIF-1α protein resulting in increased HIF-dependent gene expression (Fig. 4). PHD2 is considered the predominant hydroxylase mediating the degradation of HIFα proteins [2]. Thus, it remains to be studied whether the LPS-boosted *PHD2* accumulation can act through a negative feedback mechanism on HIFα proteins [17, 40]. The other studied HIF target *GLUT-1* was found to be significantly downregulated upon challenge with LPS in whole blood cells but increased with the subsequent hypoxic stimulus (Fig. 3). Potentially, this response contributes to changes in energy metabolism as previously proposed [24, 31, 33, 51]. Interestingly, *GLUT-1* expression was upregulated in both groups at 24 h compared to baseline conditions (Fig. 3). This differential response of HIF target genes to an inflammatory stimulus, which increases HIF1A mRNA and hypoxic activation of *HIF-1*, highlights the complexity in the regulation of HIF-1 as a transcription factor complex, controlled by hypoxia and inflammation [43, 48].

LPS treatment slightly increased the expression levels of the inflammatory cytokine *TNFA* in blood cells after 2 h. This rapid and acute immune activation aligns with findings from human studies utilizing the endotoxemia model, underscoring the systemic inflammatory response to LPS [1, 36, 38]. Interestingly, this LPS induction was affected by hypoxic priming, showing the increased upregulation of TNFA mRNA after exposure to hypoxia. In contrast, *IL6* was substantially increased by in vivo exposure to LPS under normoxia but remained at basal levels after hypoxic priming, i.e. exposure to hypoxia before LPS injection significantly dampened *IL6* expression.

Based on our findings, we anticipate alterations in protein expression, leading to subsequent effects on cellular behaviour, as evidenced by the observed rapid adaptation at the RNA level and enduring effects on mRNA levels (Fig. 3: 24 h: *HIF-1A*, *GLUT*, *ADM*; Fig. 4: *PHD2*). While there remains potential for translational regulation, our analysis indicates that for the genes investigated, changes in protein levels typically mirror alterations in transcription. However, our current study protocol has not permitted the demonstration of any discernible effects on the immune phenotypic behaviour or metabolic adaptation of the cells. Future investigations may incorporate extended observation periods or ex vivo challenge of immune cells in vitro to elucidate these aspects further.

Taken together, these observations indicate that in vivo hypoxia with 10.5% oxygen is sufficient to have an impact on inflammatory induced gene expression and potentially modulates immune reaction of whole blood cells and especially leukocytes. We assume that the RNA extracted from whole blood samples of subjects predominantly originates from leukocytes, with only a minimal fraction stemming from reticulocytes or red blood cells since red blood cells are lysed at the beginning of the blood sampling before RNA isolation and numerous publications demonstrate gene expression of leukocytes after PAXgene® RNA isolation. Remarkably, however, previous research indicates that mature red blood cells primarily contain microRNAs and mRNA transcripts associated with erythrocyte differentiation and function [10]. This supports the assumption that our observed changes in gene expression are primarily influenced by the abundance of leukocyte RNA in the sampled whole blood [10].

Numerous critical and chronic illnesses, including sepsis, acute respiratory distress syndrome, chronic obstructive pulmonary disease and sleep apnea, are linked to hypoxaemia with an inflammatory component [44]. The mechanisms involved in oxygen sensing and hypoxia signalling represent potential therapeutic targets for managing those and inflammatory diseases [12]. A substantial endeavour has been dedicated to the development of pharmacologic activators of HIF through the inhibition PHDs, commonly referred to as prolyl hydroxylase inhibitors (PHIs) [25, 52, 57]. These drugs have entered clinical use to ameliorate anaemia due to EPO deficiency. EPO is a classical HIF target, and therapy with PHIs successfully aims at increasing HIF-dependent expression in EPO-producing renal cells. However, leukocytes will also react to PHIs with HIF accumulation such as under hypoxia. It remains to be studied whether PHI treatment will have similar effects as hypoxic priming of leukocytes.

### Limitations

Evidence suggests that high altitude-hypoxia affects blood leukocytes, for example, the redistribution of circulating T cells, but not B lymphocytes [14, 41, 42]. Additionally, reduced cytokine levels following prolonged exposure to high-altitude hypoxia over the course of days to weeks, particularly under unstimulated baseline conditions, have been reported [14, 41]. Controversially, in other studies, increased IL-6 levels were measured upon hypoxia in human blood, which was interpreted as facilitating angiogenesis through the induction of *VEGF* and *EPO* [27, 34, 39]. In the present study, we were, however, neither able to provide protein data from blood leukocytes nor changes in the immune cell phenotypic behaviour influenced by hypoxia.

When comparing our in vivo data to human high-altitude studies, it is important to consider that we specifically designed our study to explore acute short-term effects, with a maximum observation duration of 24 h. Consequently, our findings may not be directly comparable to those from studies conducted over weeks in hypobaric hypoxia.

Taken together, hypoxia and inflammation are integrated and present a multi-layered interplay, which depends highly on the order of occurring stimuli. Not only acute hypoxia but also HIF-increasing PHIs have the potential to shape immune reactions and immune cell–specific responses. Further investigations will elucidate this interplay in more detail on a molecular basis to better understand pathologies with inflammatory hypoxia.

### Supplementary Information

Below is the link to the electronic supplementary material.

Supplementary file1 (DOCX 406 KB)

## Data Availability

The data sets generated during and/or analysed during the current study are available from the corresponding author on reasonable request.
