# Supplementary material for: Exposure to normobaric hypoxia shapes the acute inflammatory response in human whole blood cells in vivo
Source: Pflugers Arch. 2024 May 7;476(9):1369–81. doi: 10.1007/s00424-024-02969-2 (PMC11310243; doi:10.1007/s00424-024-02969-2)
Supplement: Supplementary file 1 — Supplementary file1 (DOCX 406 KB) [file 424_2024_2969_MOESM1_ESM.docx]

**Supplemental material**


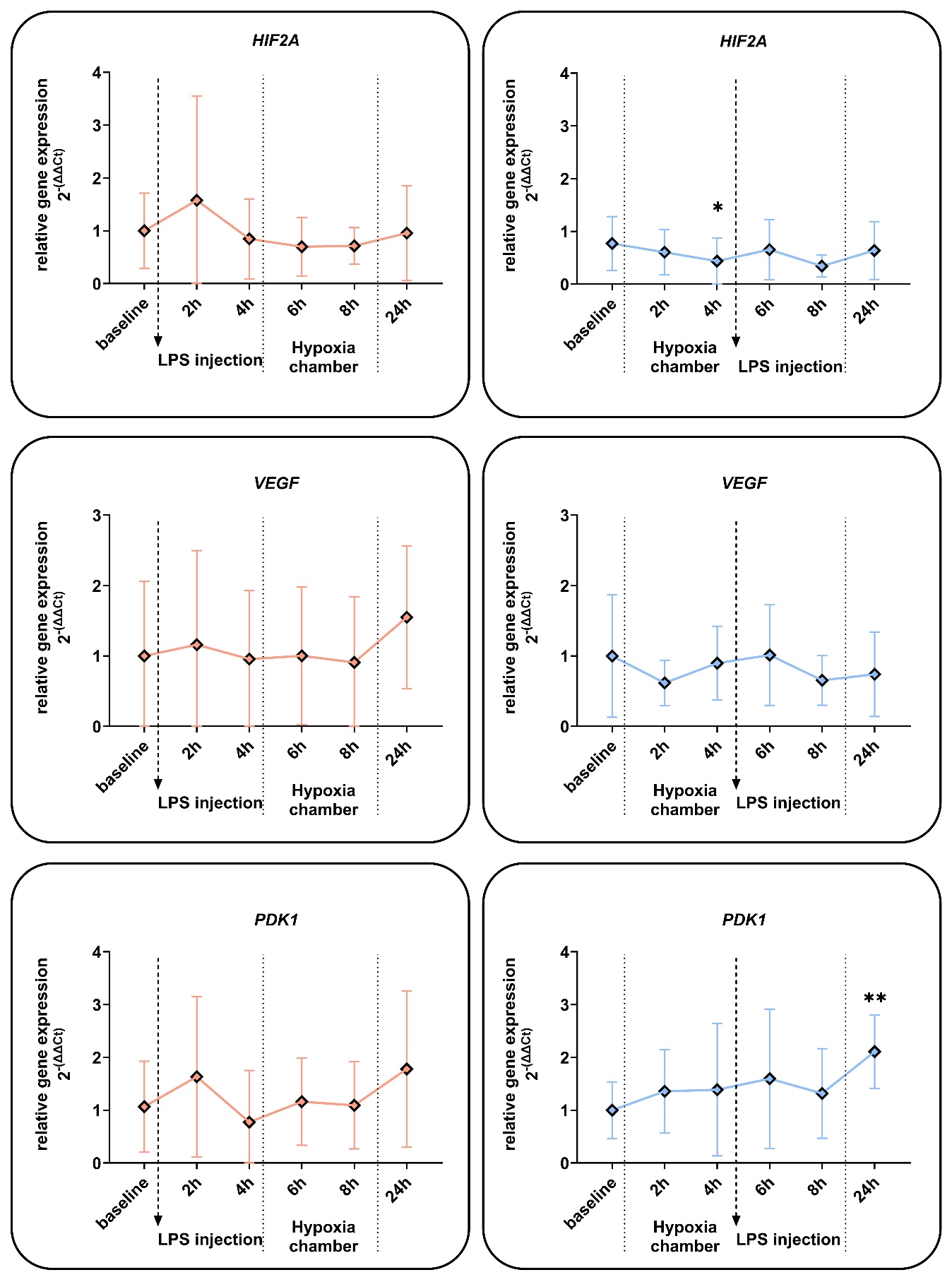


Supplemental Figure: Effects of hypoxia and LPS on HIF2A and HIF-related gene expression of whole blood cells

Relative gene expression of whole blood cells, from blood samples of human subjects that were treated with a single *E. coli* lipopolysaccharide (LPS) injection (0.4 ng/kg) and a 4-hour phase of exposure to hypoxia (normobaric, 10.5 % O2) prior or after LPS injection. Shown are mRNA expression fold changes from hypoxia-inducible factor 2α (*HIF2A*), vascular endothelial factor (*VEGF*) and pyruvate dehydrogenase kinase 1 (*PDK1*). Expression levels of mRNA were normalised to *ACTB* and are presented as 2-(ΔΔCT) values (mean ± SD, mixed-effects analysis with repeated measures and Tukey´s or Sidak´s multiple comparisons test; * = p < 0.05, ** = p < 0.01, n = 8 - 13).

Supplementary Table: Primer sequences of specific PCR products used for RNA quantifications of blood samples via RT-PCR.

| Target gene | Primer | sequence |
| --- | --- | --- |
| *ACTB* | 3´ | CAGCGGAACCGCTCATTGCCAATGG |
|  | 5´ | TCACCCACACTGTGCCCATCTACGA |
| *HIF1A* | 3´ | CTCCATTACCCACCGCTGAA |
|  | 5´ | TCACTGGGACTATTAGGCTCAGGT |
| *HIF2A* | 3´ | CGGAGGTGTTCTATGAGCTGG |
|  | 5´ | AGCTTGTGTGTTCGCAGGAA |
| *GLUT-1* | 3´ | CTAGCGCGATGGTCATGAGT |
|  | 5´ | TCTGGCATCAACGCTGTCTT |
| *ADM* | 3´ | AGTCGTGGGAAGAGGGAACT |
|  | 5´ | ATCCGGACTGCTGTCTTCGG |
| *PDK1* | 3´ | TGAACGGATGGTGTCCTGAG |
|  | 5´ | GGCCAGGTGGACTTCTACG |
| *VEGF-A* | 3´ | CCGCCTCGGCTTGTCACA |
|  | 5´ | GCAAGACAAGAAAATCCCTGTGGGCC |
| *PHD1* | 3´ | TGGCCCTGGACTATATCGTG |
|  | 5´ | GGCACCAATGCTTCGACAG |
| *PHD2* | 3´ | GCACGACACCGGGAAGTT |
|  | 5´ | CCAGCTTCCCGTTACAGT |
| *PHD3* | 3´ | CACAGCGAGGGAATGAACCT |
|  | 5´ | TCCTGCTGTTAAGGCTTCCG |
| *IL6* | 3´ | CCACTCACCTCTTCAGAACGAA |
|  | 5´ | TGCATCTAGATTCTTTGCCTTTTT |
| *TNFA* | 3´ | CTCAGCTTGAGGGTTTGCTAC |
|  | 5´ | TGCACTTTGGAGTGATCGGC |
